# Lipid Modification and Membrane Localization of Proteins in Cell-Free System

**DOI:** 10.1021/acssynbio.5c00155

**Published:** 2025-06-19

**Authors:** Rena Matsumoto, Tatsuya Niwa, Kaori Kuno, Yasuhiro Shimane, Yutetsu Kuruma, Takashi Kanamori

**Affiliations:** † GeneFrontier Corporation, 273-1 Kashiwa, Kashiwa, Chiba 277-0005, Japan; ‡ Cell Biology Center, Institute of Integrated Research, Institute of Science Tokyo, Yokohama, Kanagawa 226-8501, Japan; § Institute for Extra-Cutting-Edge Science and Technology Avant-Garde Research (X-star), 13570 Japan Agency for Marine-Earth Science and Technology (JAMSTEC), 2-15 Natsushima-cho, Yokosuka, Kanagawa 237-0061, Japan

**Keywords:** cell-free protein synthesis, PURE system, liposomes, lipid modification, VHH antibody

## Abstract

Post-translational modifications are an essential process for proper protein function and localization. In particular, lipid modification plays a crucial role in the spatial regulation of proteins functioning on a lipid membrane surface. While cell-free protein synthesis allows rapid protein production, technical advances in lipidation modification are behind. Here, we developed a cell-free system for the myristoylation and palmitoylation of proteins. Based on our previous study, we improved myristoylation efficiency by trimming a precursor nascent peptide, which undergoes lipidation at the N-terminal glycine. We also found that N-myristoyltransferase (NMT) catalyzes both myristoylation and palmitoylation. The localization of lipidated proteins onto liposomes is further aided by the insertion of polyarginine residues downstream of the NMT recognition site. Finally, we demonstrated that lipidation of VHH antibodies and localization onto liposomes resulted in target-specific binding to cancer cells. This system offers a platform for displaying soluble proteins on lipid membranes, with potential applications in developing liposomes for targeted cell binding.

## Graphical abstract

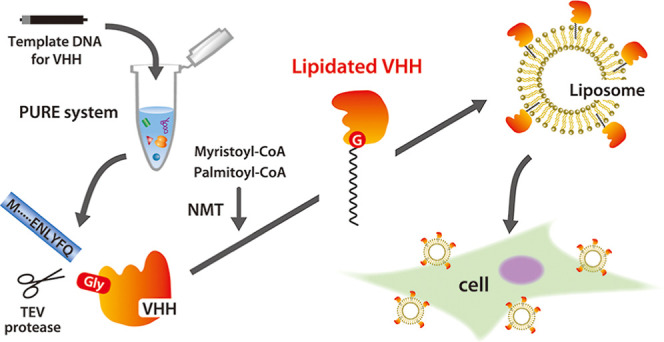

## Introduction

Cell-free protein synthesis systems, which are also known as transcription-translation systems, are powerful tools for obtaining proteins of interest rapidly and in parallel.^1–3^ In particular, a reconstructed cell-free system, the PURE system,^4^ which is composed of all purified translational factors and ribosomes, has the advantage of being easily customizable; therefore, it has been applied in many research fields, including drug discovery research.^5–10^ Within the PURE system, nascent polypeptides synthesized by ribosomes mostly fold autonomous to adopt the proper conformation according to Anfinsen’s dogma.^11^ This process occurs not only post-translationally but also during the translation.^12–14^ For proteins that cannot fold autonomously, the addition of chaperones in the PURE system enhances the solubility in some proteins.^15^ When synthesizing membrane proteins, small-sized lipid membrane vesicles (liposomes) or disc-shaped lipid bilayers (nanodiscs) are supplemented and used as a localization place of the synthesized membrane proteins.^16,17^ So membrane localized proteins are able to maintain enzymatic activities^18^ or complex formation with partner proteins.^17,19^ Therefore, the PURE system allows not only for simple synthesis of proteins but also for control of the properties and localization of the synthesized proteins by customizing the reaction conditions. Limitations of the PURE system, however, stand as the next challenges, for example, protein modifications such as glycosylation, acetylation, phosphorylation, and lipidation.

The post-translational modifications at the N-terminus of nascent polypeptides are important events to regulate protein stability, functionality, subcellular localization, quality control, and signal transduction.^20,21^ Protein lipidation, such as myristoylation or palmitoylation, is particularly important because it is involved in higher-order protein functions related to the cell membrane and intracellular organelles. Once translated, polypeptides are subjected by methionine aminopeptidase (MAP) to cut off the initial methionine and expose glycine that will be subsequently modified for lipidation by enzymes such as N-myristoyltransferase (NMT). Lipidated polypeptides therefore acquire a hydrophobic property, promoting their localization onto intracellular membrane organelles.

In the previous study, we developed the PURE system for implementing post-translational modifications.^22^ Cell-free synthesized superfolder green fluorescent protein (sfGFP) was processed with MAP and subsequently bound to myristoyl chains by NMT, which was also produced in another PURE system. We also observed the effect of myristoylated sfGFP inside cell-size membrane vesicles; the myristoylated sfGFP localized onto the vesicle membrane and showed fluorescence at the edge of the vesicles. Although we have developed the basis for cell-free protein lipidation, the problem remained that only up to 20% of the substrate protein was lipidated. This low efficiency is due to insufficient methionine digestion by MAP, so most synthesized proteins are not available for myristoylation. To overcome this, we redesigned the initial steps of cell-free protein lipidation and tried to improve the efficiency. Additionally, we also tested the binding of a longer acyl chain, which confers greater hydrophobicity at the N-terminal of proteins. Finally, we applied our cell-free system to lipidate VHH antibodies and immobilize them on a small-size liposome membrane. The PURE system, we developed in this study, can lipidate proteins and immobilize them on lipid membranes and therefore may be a new tool for the rapid production of drug-delivery liposomes that specifically bind to target cells.

## Results

### New Design for N-Terminal Myristoylation of Cell-Free Synthesized Proteins

First, we designed the construct of the substrate protein that will be lipidated by NMT at the N-terminus (Figure 1A). We placed the NMT recognition motif (GSTLSAE), which originated from the N-terminal sequence of Goα protein, at the upstream of the sfGFP. The serine residue at the second amino acid of Goα has been replaced from the original cysteine,^22^ so we named this construct Goα­(S)-sfGFP. At the upstream of Goα­(S)-sfGFP, we introduced methionine-lysin-isoleucine (MKI) followed by six histidine with SSG-linker and a TEV protease-recognition sequence (ENLYFQG). The MKI contributes to improving protein synthesis efficiency by reducing the GC content at the initial nucleotide sequence.^23^ Unlike the original design, here we utilize TEV protease digestion, leading to exposure of the N-terminal glycine residue essential for acyl chain attachment by NMT. The substrate Goα­(S)-sfGFP and NMT were individually synthesized using the PURE system, where TEV protease was supplemented in the substrate side (Figure 1B). The resulting mixtures were mixed in the presence of an acyl chain donner for protein lipidation.

**Figure 1:**
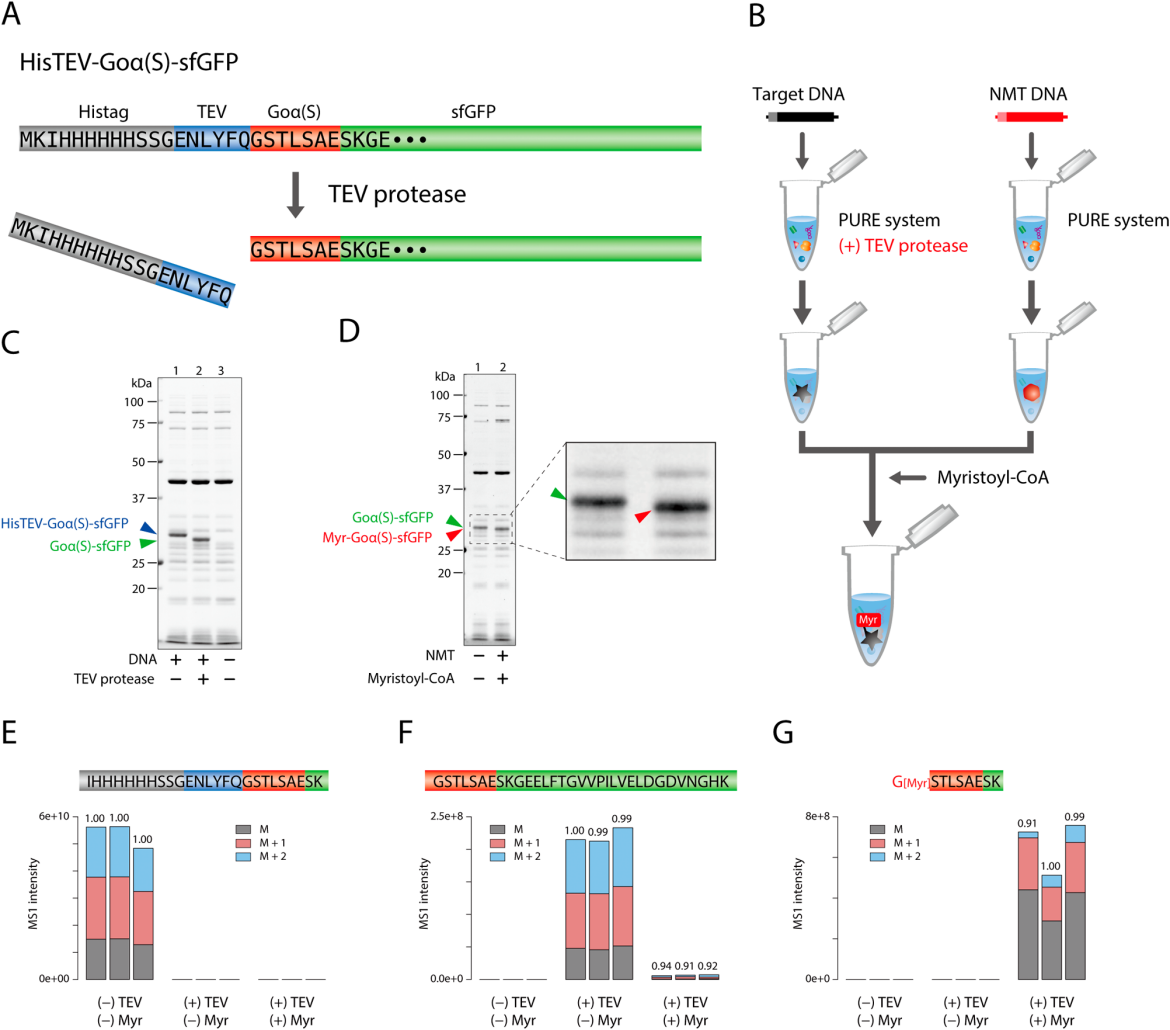
Lipid modification of cell-free synthesized proteins. (A) The construct of a substrate protein for lipid modification. (B) A schematic of TEV digestion of substrate protein and myristoylation by NMT. (C) SDS-PAGE analysis of TEV-digested substrate protein. HisTEV-Goα­(S)-sfGFP was synthesized in the presence or absence of TEV protease and analyzed by SDS-PAGE. The bands of the digested and not digested products are indicated by green and blue arrowheads, respectively. (D) SDS-PAGE analysis of myristoylated protein. Goα­(S)-sfGFP and the myristoylated Goα­(S)-sfGFP are indicated by green and red arrowheads, respectively. (E) Extracted ion chromatograms of N-terminal peptide derived from HisTEV-Goα­(S)-sfGFP after trypsin digestion (IHHHHHHSSGENLYFQGSTLSAESK, *M* = 947.4464 Da, *M* + 1 = 947.7807 Da, *M* + 2 = 948.1149 Da, *z* = 3). (F) Extracted ion chromatograms of N-terminal peptide derived from TEV-digested Goα­(S)-sfGFP after trypsin digestion (GSTLSAESKGEELFTGVVPILVELDGDVNGHK, *M* = 1099.8998 Da, *M* + 1 = 1100.2341 Da, *M* + 2 = 1100.5684 Da, *z* = 3). (G) Extracted ion chromatograms of N-terminal peptide derived from myristoylated HisTEV-Goα­(S)-sfGFP after trypsin digestion (G­[Myr]-STLSAESK, *M* = 545.3237 Da, *M* + 1 = 545.8252 Da, *M* + 2 = 546.3266 Da, *z* = 2). (E–G) Three bars represent three independent measurements. The number above the bar indicates an isotope dot product (idotp) score. The retention time was confirmed by the peptide search results from the MS/MS spectral search.

The cell-free synthesis and TEV-digestion were confirmed by SDS-PAGE analysis, showing almost all substrate proteins were trimmed (Figure 1C). The previous design used MAP to remove the initial methionine and expose glycine. However, due to the insufficiency of methionine removal by MAP, we could not obtain high efficiency of the myristoylation, less than 20%. The current design could improve this point by utilizing TEV protease so that more efficient lipidation is expected. To prove this, we performed acyl chain transfer to the TEV-digested Goα­(S)-sfGFP by mixing it with the cell-free synthesized NMT and acyl chain donor, myristoyl-CoA. An SDS-PAGE analysis showed a slight band shift of Goα­(S)-sfGFP to the lower molecular side when NMT and myristoyl-CoA were presented, indicating that the substrate protein was myristoylated (Figure 1D). This was further confirmed by observing a significant decrease of GSTLSAESKGEELFTGVVPILVELDGDVNGHK fragment and the appearance of G­[Myr]­STLSAESK fragment by mass spectrometry analysis, showing almost all the TEV-digested protein was myristoylated (Figure 1E–G). By using a newly designed substrate protein and TEV protease, we could improve the lipidation efficiency from ∼40% to ∼95% compared to the previous version.^22^

### Membrane Localization of Myristoylated-GFP Inside Giant Vesicles

The hydrophobic property of the myristoylated protein was evaluated by performing a myristoylation reaction inside giant unilamellar vesicles (GUVs) composed of 80 mol % POPC and 20 mol % POPG. The TEV-digested GFP and NMT were encapsulated inside GUVs together with myristoyl-CoA to facilitate myristoylation and membrane localization of the resulting sfGFP. Here, to mimic the molecular crowding effect in the cytoplasm, we also encapsulated Ficoll PM70, a bulky molecule that does not affect the internal reaction but is expected to promoting the membrane localization by depletion effect. We observed the localization of sfGFP fluorescence on the GUV membrane after 2 h of reaction (Figures 2A and S1). This localization was more evident after 20 h of reaction. Membrane localization efficiency was determined by measuring fluorescent intensity at the edge of GUV and lumen (Figure 2F). Because membrane localization efficiency is affected by the size of GUV even in the same sample (Figure S2), we normalized the obtained values by dividing them by the specific surface area (SSA). No membrane localization was observed when TEV protease or NMT was omitted (Figures 2B,C, and S1), suggesting that only myristoylated sfGFP localized onto the GUV membrane. We also tested whether the presence of Ficoll PM70 contributes to membrane localization efficiency or not. When the crowding effect by Ficoll PM70 was omitted, the membrane localization efficiency was reduced (Figures 2D,F and S1). In summary, the membrane localization efficiency improved from 70% membrane localization/SSA in the previous study^22^ using MAP to 140% membrane localization/SSA in the present study using TEV and Ficoll PM70 (Figures 2E,F and S1).

**Figure 2:**
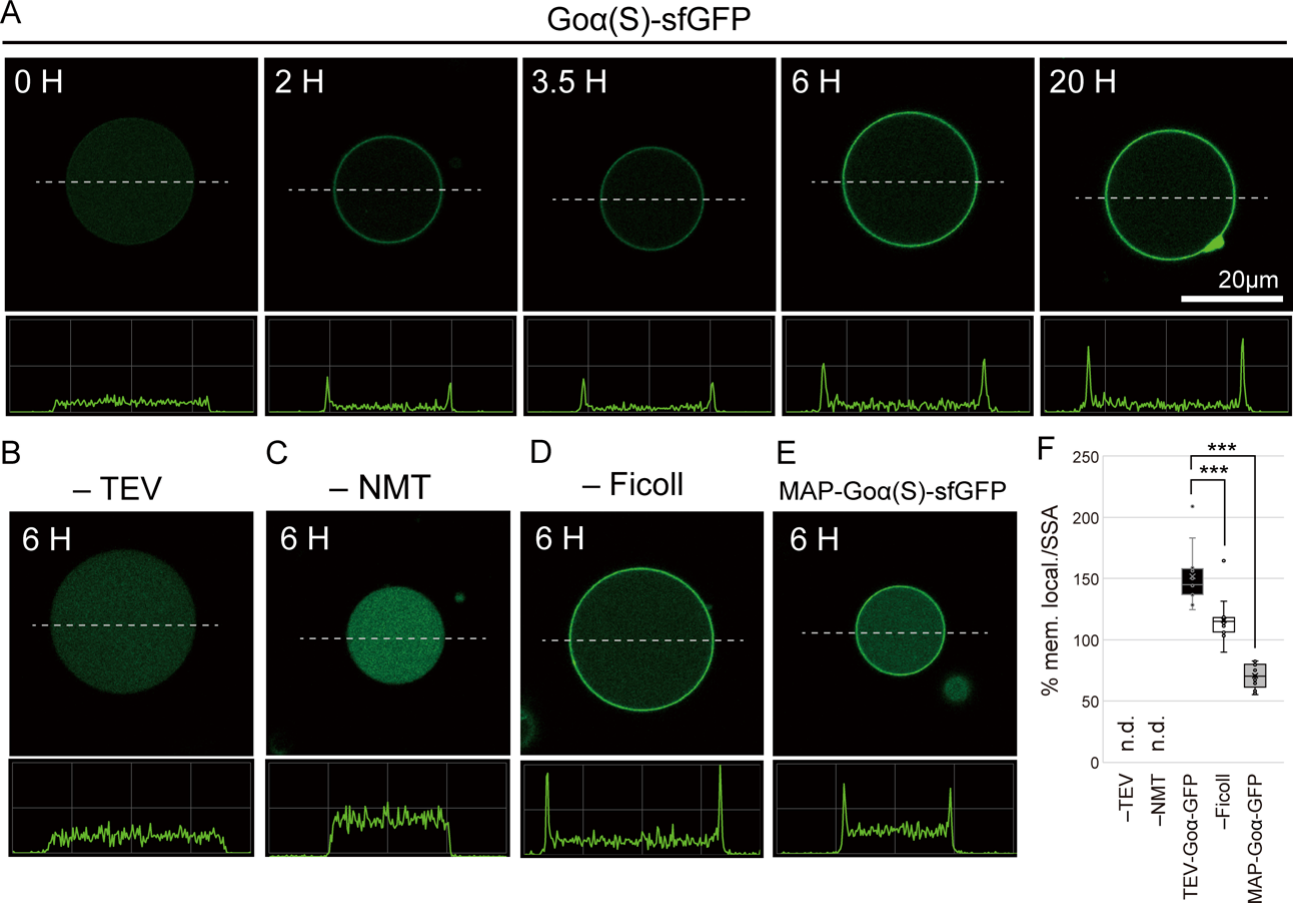
Protein lipid modification and membrane localization within giant vesicles. (A) Myristoylation of Goα­(S)-sfGFP inside giant vesicles. The reacted vesicles were observed at the indicated time. The vesicles, where lacking TEV (B), NMT (C), and Ficoll (D) were also observed at the indicated time. The Goα­(S)-sfGFP protein prepared by using MAP instead of TEV was also tested myristoylation inside giant vesicles (E). (F) The membrane localization efficiencies, which were normalized with SSA, of (B), (C), (A (6H)), (D), and (E) were compared by analyzing 10 vesicles for each sample. Welch’s *t*-test were performed, where *** indicates <0.0005. The fluorescent intensities on the membrane were measured by plot profile (ImageJ) and shown as graphs under the vesicles. Bar size: 20 μm.

### Introduction of Polyarginine Residues Promotes Localization to Nanodisc Membrane

By revising the experimental setup, we could improve the lipidation efficiency and show protein localization onto the GUV membrane. From the viewpoint of application of this technology, however, it is more valuable to localize proteins onto the outer surface of small-size liposome membranes for the use of, for example, a target-specific liposome delivery system. With such motivation, we next experimented to see the localization of myristoylated sfGFP onto the liposome membrane. Different from the experiments using GUVs, the substrate Goα­(S)-sfGFP is lipidated at the exterior of the liposome and localized onto the liposome surface. Myristoylation of the substrate Goα­(S)-sfGFP was performed in the presence of liposomes. However, unexpectedly, the myristoylated sfGFP was not localized onto the liposome membrane, unlike the GUV membrane (Figure S3). We speculate that the difference in membrane curvature between the 30 μm diameter GUV (*H* = −6.7 × 10^4^ m^–1^, inside) and the 100 nm diameter liposome (*H* = 2.0 × 10^7^ m^–1^) may contribute to the observed differences in localization.

To promote protein localization onto the liposome membrane, we reconsidered the design of the substrate protein. We inserted three (R3) or six (R6) arginine residues to confer a positive charge between Goα and sfGFP, expecting to strengthen the electrostatic interaction between protein and membrane surface^24,25^ (Figure 3A). Before testing with liposomes, the effect of the addition of arginine residues was evaluated using a nanodisc, which is a disc-shaped lipid membrane composed of 100 mol % DMPG or DMPC. Using the Goα­(S)-sfGFP purified and TEV-digested in advance, myristoylation was performed in the presence of nanodiscs (Figure 3B). At this time, in addition to myristoyl-CoA, palmitoyl-CoA was also tested as a possible donner of lipidation. Although the NMT used in this study has a dominant specificity for myristoyl-CoA, it is known to also accept palmitoyl-CoA as a substrate even if the activity is low.^26^ Mass spectrometry analysis could not detect palmitoylated peptides in the gel-digested samples, probably due to their strong hydrophobicity. However, when we changed the preparation procedure to in-solution digestion after urea denaturation, efficient palmitoylation to the Goα­(S)-sfGFP was confirmed after the lipidation reaction using palmitoyl-CoA (Figure S4).

**Figure 3:**
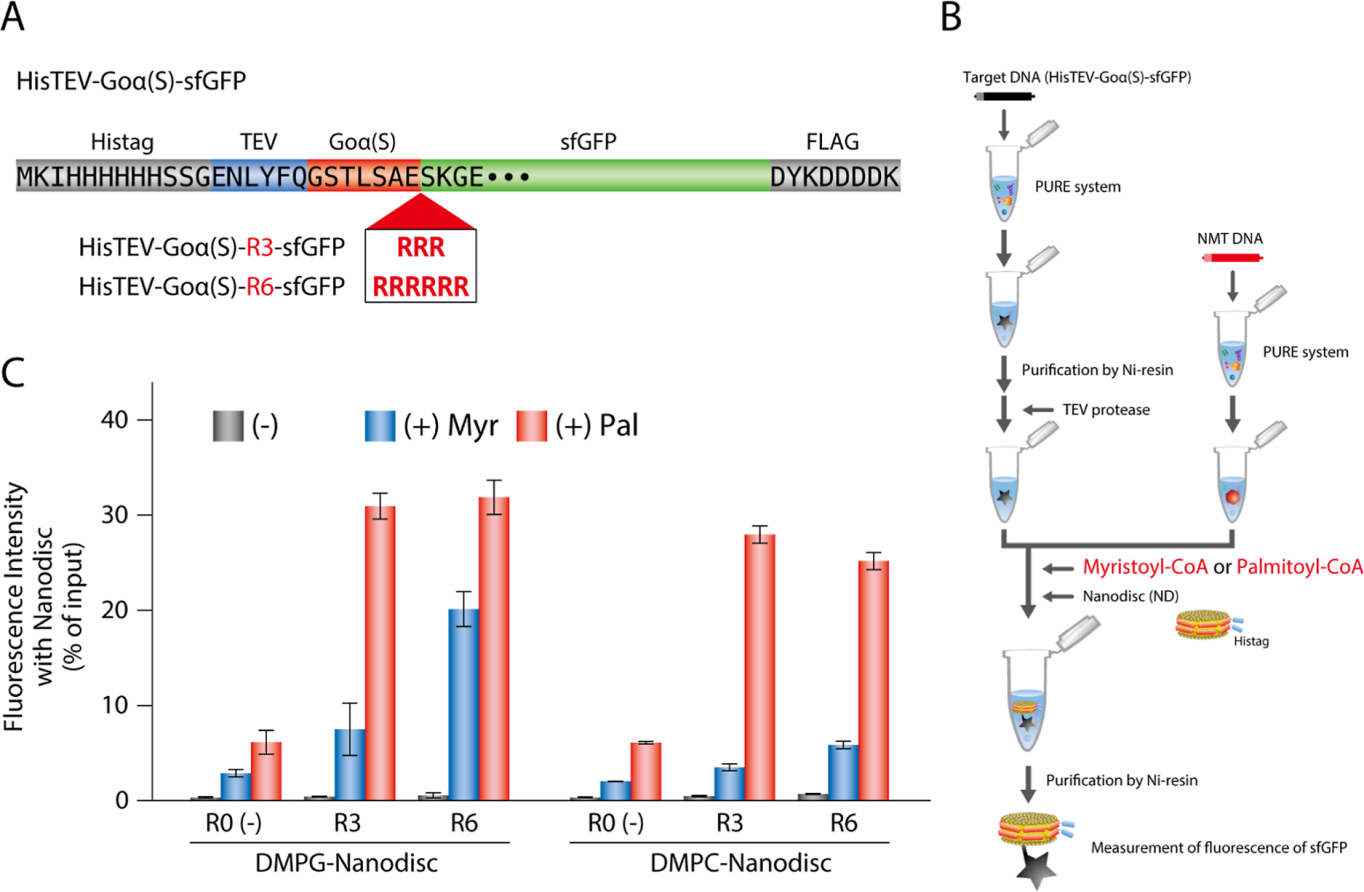
Lipid modification of polyarginine-introduced proteins. (A) The construct of substrate proteins containing three (R3) or six (R6) polyarginine residues downstream of Goα­(S) motif. (B) A schematic of lipid modification and integration to nanodisc. The substrate protein synthesized by PURE system was purified and trimmed by TEV protease. Lipid modification was performed with myristoyl- or palmitoyl-CoA in the presence of nanodisc. The resulting nanodisc was purified and analyzed for colocalization of lipidated proteins. (C) Co-collection of protein with nanodisc containing DMPG (DMPG-nanodisc) or DMPC (DMPC-nanodisc). The fluorescent intensities of Goα­(S)-sfGFP in the purified nanodisc fraction were measured and shown as graph. The lipid modifications were carried out for all types of protein (R0, R3, and R6) in the absence or presence of myristoyl-CoA (Myr) or palmitoyl-CoA (Pal). Average of fluorescent intensity and standard deviations were derived from three independent experiments.

When myristoylation was performed using the construct without the arginine insertion (R0), only 2.0% or 2.8% of the input Goα­(S)-sfGFP was coisolated with nanodisc containing DMPC or DMPG, respectively, suggesting almost no membrane localization to the nanodisc (Figure 3C). In contrast, 3.5% and 5.9% (with DMPC) or 7.5% and 20.1% (with DMPG) of myristoylated Goα­(S)-sfGFP were coisolated with nanodiscs containing R3 and R6, respectively (Figure 3C). As expected, the insertion of arginine residues was shown to be effective in increasing the membrane localization efficiency. The effect of the arginine number between R3 and R6 was significant in the case of myristoylated Goα­(S)-sfGFP and nanodisc-containing DMPG, indicating the importance of electrostatic interactions between lipidated proteins and the membrane surface. Surprisingly, when palmitoyl-CoA was used, a higher amount of Goα­(S)-sfGFP was collected in the nanodisc fraction for all three constructs with the efficiency of 6.1%, 28.0%, and 25.2% (with DMPC) or 6.1%, 30.9%, and 31.9% (with DMPG) for R0, R3, and R6, respectively (Figure 3C). The insertion of arginine residues and the use of palmitoyl-CoA resulted in a more than 10-fold increase in membrane localization compared to that of R0 using myristoyl-CoA. In the case of palmitoylated Goα­(S)-sfGFP, there was no difference in membrane localization efficiency between R3 and R6 insertion. This suggests that the palmitoyl chain was predominant because it provides sufficient hydrophobicity for the nanodisc membrane localization. And a negative membrane charge affects myristoylated Goα­(S)-sfGFP. These results indicate that the addition of positive charges and the lipidation promote the localization of proteins to lipid membranes, as designed.

### Localization onto Liposome Membrane

We performed the same lipidation experiment in the presence of liposomes and confirmed that the lipidated Goα­(S)-sfGFP is localized on the liposome membrane (Figure 4A). The liposomes were composed of POPC/POPG/DSPE-PEG2000/rhodamine-DHPE (68:30:2:0.5 mol %). The resulting liposomes were fractionated into six fractions, including the precipitant, by ultracentrifugation with OptiPrep density gradation, where the fractions 1–2 from the top contain liposomes. In the case of Goα­(S)-sfGFP with R3, a large part of the proteins remained at the bottom side (Figure S5), indicating less efficiency of membrane localization of Goα­(S)-sfGFP­(R3). On the other hand, Goα­(S)-sfGFP with R6 was found in the liposome fractions (Figure 4D). To analyze NMT dependency, we tested the lipidation reaction with several amounts of the NMT. When palmitoyl-CoA was used as an acyl donner, about 60–70% of Goα­(S)-sfGFP­(R6) was collected in the liposome fractions when 8.5, 17, or 34 μL of the NMT-expressing PURE system was used for a 40 μL reaction (Figure 4B,D). Similarly, about 70% of Goα­(S)-sfGFP­(R6) was found in the liposome fractions when 17 μL of NMT-expressing PURE system and myristoyl-CoA was used (Figure 4C). Lower membrane localization efficiency was found when 8.5 or 34 μL of the NMT-expressing PURE system was used (Figure 4C). In all cases, no membrane localization was observed when NMT was omitted (Figure 4D), suggesting that only lipidated proteins are capable of localizing onto liposomes and that the positive charge of R6 alone is not enough for the localization. These results show that the R6 introduced substrate protein can localize onto the liposome membrane by lipidation with a palmitoyl or myristoyl chain.

**Figure 4:**
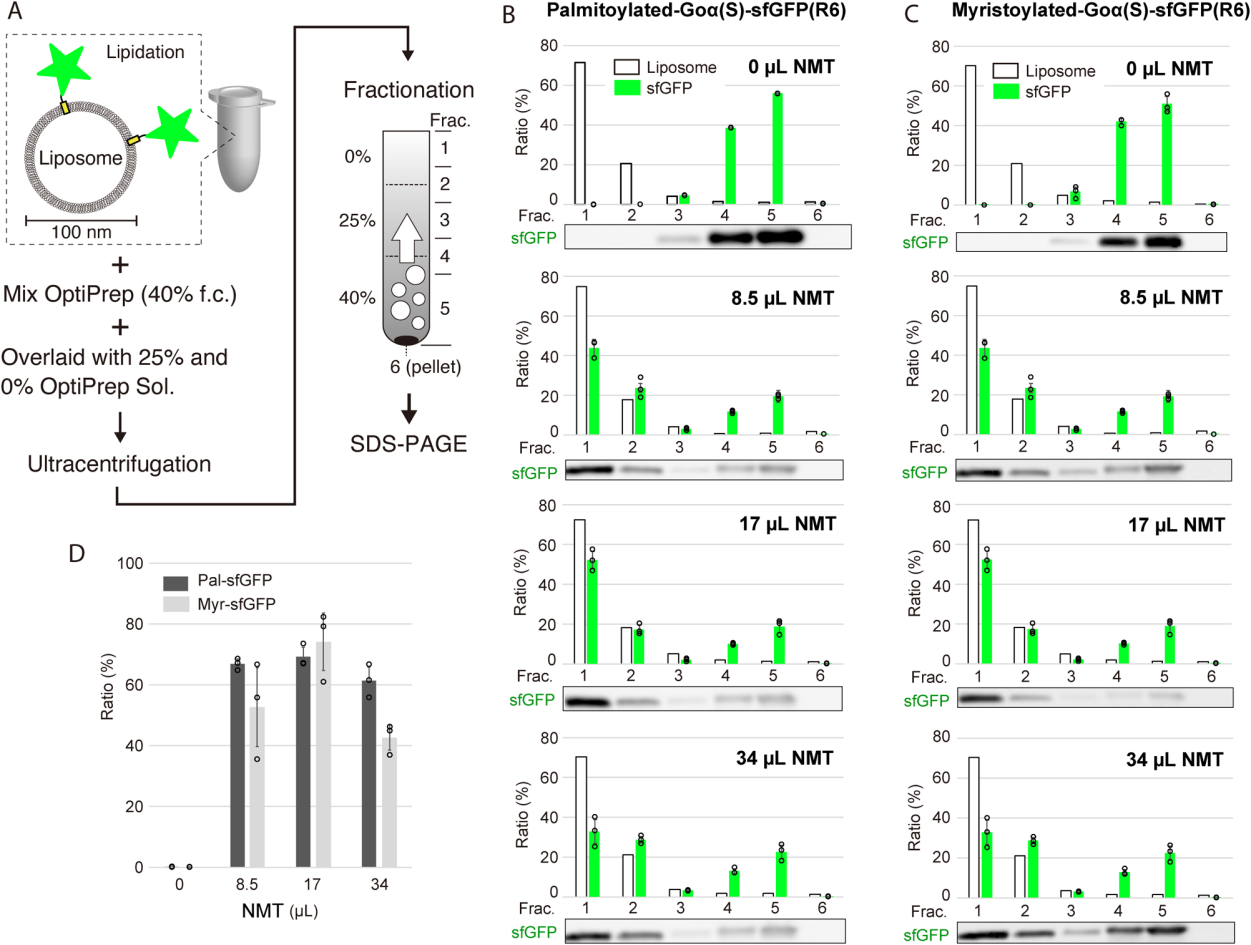
Localization of lipidated protein onto liposomes. (A) A scheme of flotation assay. Lipid modification of Goα­(S)-sfGFP was carried out in the presence of liposomes. The resulting liposomes fractionated by ultracentrifugation with OptiPrep density gradient. Collected fractions were analyzed by SDS-PAGE to confirm the presence of protein. Goα­(S)-sfGFP containing R6 was tested in palmitoylation (B) or myristoylation (C), along with the series of NMT input volumes (0, 8.5, 17, and 34 μL). The fractionation pattern of Goα­(S)-sfGFP (green color bar) and liposome (white color bar) are shown with an example of gel images below the graph. (D) Membrane localization efficiency of palmitoylated- or myristoylated-Goα­(S)-sfGFP­(R6) with varied NMT amounts. For all data, average of fluorescent intensity and standard deviations were derived from three independent experiments.

In the experiments using a nanodisc, there was a difference in membrane localization efficiency between palmitoylated and myristoylated GFP (Figure 3C), whereas no significant difference was observed in the case of liposome (Figure 4D). This discrepancy may be attributed to the difference in membrane surface area. Compared with nanodisc, the continuous lipid membrane of liposome provides a larger surface area, which likely increases the frequency of contact even for the less hydrophobic myristoyl moiety.

### Localization of Lipidated-VHH Antibody onto Liposome Membrane

Lastly, we synthesized the VHH antibodies and localized them onto the liposome membrane by simply replacing the sfGFP gene with the anti-HER2 VHH gene.^27^ We synthesized three types of VHH antibodies (Figure S6), i.e., without arginine and with polyarginine (R3 and R6). The synthesized proteins were trimmed by TEV (Figure 5A) in all types, as in the case of sfGFP (Figure 1C). The resulting VHH protein was named Goα­(S)-VHH. The Goα­(S)-VHH was then acylated with palmitoyl-CoA or myristoyl-CoA. We could not detect the band shift of the palmitoylated-VHH on the SDS-PAGE, but we could clearly observe the band shift of the myristoylated-VHH (Figure 5B), again the same as that of sfGFP (Figure 1D). Next, Goα­(S)-VHH was acylated with palmitoyl-CoA in the presence of liposomes with the same lipid composition as in Figure 4. The resulting reaction mixture was fractionated by a floating assay. Similar to Goα­(S)-sfGFP, palmitoylated Goα­(S)-VHH­(R6) was collected in the liposome fractions (Figure 5C). Here, good membrane localization efficiency was observed also for Goα­(S)-VHH­(R3), unlike for Goα­(S)-sfGFP­(R3) (Figure S5). The isoelectric points of sfGFP and VHH are 6.34 and 6.57, respectively (calculated by SnapGene), so this difference is likely due to the properties of the protein, such as the structure of the protein. No VHH antibody protein was collected in the liposome fractions when the liposomes were not supplied (Figure S7). Approximately 90% of Goα­(S)-VHH­(R6) and 65% of Goα­(S)-VHH­(R3) were membrane localized. By quantifying Goα­(S)-VHH­(R6), we estimated that about 1500 or 2200 VHH molecules were localized on a liposome in fraction 1 or 2, respectively. These correspond to protein-to-lipid ratios of approximately 1:60 and 1:40, respectively. To examine whether the VHH antibodies localized on the liposome correctly recognize the antigen (HER2) and maintain their binding ability, we exposed the resulting liposomes to breast cancer cells expressing HER2 (Figure S8). When Goα­(S)-VHH­(R3) was synthesized and localized on the liposomes, we observed the accumulation of the resulting liposomes, which contain rhodamine-DHPE, on the HER2-expressing cells (BT474) (Figures 5D and S9). This was more significant when Goα­(S)-VHH­(R6) was lipidated. The difference of fluorescent intensities between R3 and R6 faithfully reflects the results of the flotation assay (Figure 5C). On the other hand, no accumulation of liposomes was observed when HER2 negative cells (T47D) were used (Figure 5E) or NMT was omitted (Figure S9). These results show that cell-free synthesized VHH antibodies were lipidated and localized on the liposome membrane, thereby endowing them with the ability to bind their target specifically.

**Figure 5:**
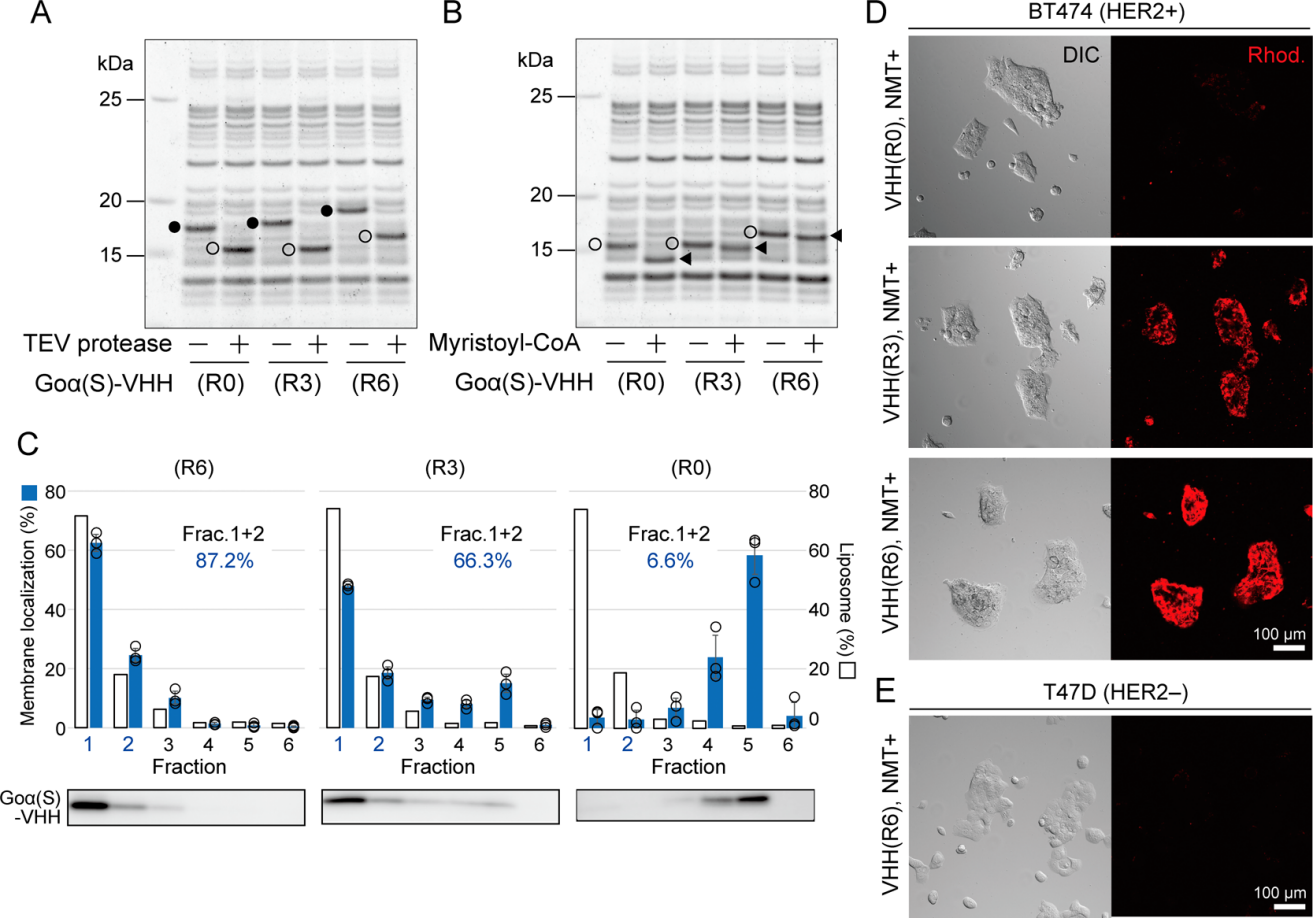
Localization of VHH antibodies onto liposomes by lipid modification. (A) Synthesis and trimming of substrate protein. Goα­(S)-VHH containing R0, R3, or R6 were synthesized by PURE system in the presence or absence of TEV protease was analyzed by SDS-PAGE. Trimmed and nontrimmed protein bands are indicated by open and closed circles, respectively. (B) Lipid modification of VHH protein. Myristoylated Goα­(S)-VHH (filled triangle) was analyzed with nonmodified Goα­(S)-VHH (open circle) by SDS-PAGE. (C) Flotation assay of palmitoylated Goα­(S)-VHH. Localization of palmitoylated Goα­(S)-VHH on the liposomes were analyzed as in Figure 4B or C. Membrane localization efficiency combining fractions 1 and 2 (Frac. 1 + 2) are shown in the graphs. Average of membrane localization efficiency and standard deviations were derived from three independent experiments. (D) Specific binding of VHH-localizing liposomes to target cancer cells. Goα­(S)-VHH (R0, R3, or R6) were palmitoylated in the presence of liposomes containing rhodamine-DHPE. The resulting liposomes were mixed with the HER2 positive cells (BT474) and observed by confocal microscopy with differential interference contrast (DIC) and 561 nm laser. HER2 negative cells (T47D) were used as negative control (E). Bar: 100 μm.

## Discussion

In this study, we designed and developed the cell-free system that is capable of modifying post-translational lipidation at the N-terminal of proteins in high efficiency. The whole process, not only the synthesis of substrate proteins but also the modification enzyme, can be carried out without using living cells, making it possible to rapidly perform and evaluate protein modification within 2 days. The prototype of this system was developed in the past study,[Bibr ref22] 2023, where protein myristoylation efficiency was limited up to 20%. This is due to the low efficiency of MAP in cleaving the initial methionine to expose glycine for myristoylation. Therefore, we reconsidered this step and changed the design using TEV protease. The newly designed construct enabled us to produce precursor proteins with the high yield motif (MKI) and trim the precursor protein with nearly 100% efficiency. The effect of this increased efficiency was recognized also by observing the membrane localization of myristoylated protein within GUVs, which exhibited a higher localization efficiency compared to the previous study. Another addition to the previous study was the insertion of a polyarginine motif. Whereas the myristoylated protein could not localize onto liposomes (Figure S3), the protein inserted polyarginine (6R) showed effective localization onto the liposome membrane (Figure 4C). Additionally, we confirmed that NMT can transfer the palmitoyl chain to protein from palmitoyl-CoA. In this way, we have improved the system and developed a cell-free system that can lipidate proteins efficiently and stably.

We performed membrane localization of proteins using a GUV, nanodisc, and liposome. All these membranes have different curvatures and lipid compositions; therefore, we cannot directly compare the obtained localization efficiencies. However, we found an obvious difference in localization efficiency between nanodisc (20–30% in Goα­(S)-sfGFP­(R6) with DMPG-nanodisc, Figure 3C) and liposomes (70–80%, Figure 4D). For the cell-free lipidation reaction, we used 2 mg (lipid)/mL nanodisc and the same concentration of liposome. But, considering only the outer leaflet of the liposome is accessible for the lipidated protein, the available concentration is only 1 mg (lipid)/mL in the liposome sample. These calculations indicate that the net amount of nanodisc membrane was higher than that of liposome. Additionally, the membrane charge of the nanodisc is also higher than that of the liposome. But even so, the localization efficiency in the use of liposomes is higher than that of nanodiscs. About this issue, we speculate that the membrane curvature mainly influences the localization of the lipidated proteins. In GUVs with low curvature, the localization efficiency drops to 30–50% (Figure S2), which supports our speculation.

Considering the useful application of our cell-free system, we performed lipid modification of VHH antibodies and localization onto liposomes. VHH protein was lipidated and localized onto liposomes, the same as sfGFP. The binding activity of the VHH-liposomes to target cells was successfully observed in clear contrast to negative control cells lacking the target antigen protein. This indicates that the VHH protein maintains the functional three-dimensional while anchoring the N-terminus of the protein on the surface of the liposome membrane. Thus, our system appears to be effective in the membrane localization of multiple types of soluble proteins. This fact implies that our system has potential applications in various areas. For example, by encapsulating siRNA inside the liposome compartment,^28^ we may be able to produce a delivery system that is capable of silencing specific genes in specific cells. Similarly, by encapsulating mRNA of a viral surface protein (like the S-protein of COVID-19), it may be possible to produce mRNA vaccines that allow only specific cells to synthesize antigen proteins.^29^ In addition to antibodies, various ligand proteins may also be present on the liposome membrane.^30^ Because we have been able to produce antigen-bound liposomes using HER2 as a model, our system can also be used to deliver anticancer drugs to cancer cells. It should be noted that, since our approach does not need to use living cells and genetic recombination steps, no application procedures are required for genetic modification experiments that sometimes take long to be approved. Taking advantage of the cell-free system, it is also well suited for high-throughput work flow. Producing functional liposomes in a highly controllable and purified system is suitable as a fundamental technology for pharmaceutical research, where the risk of contamination during the manufacturing process must be minimized. On the other hand, the weakness of this system is that lipid modification is currently only applicable at the N-terminus of protein. Therefore, it is not suitable for proteins whose function is impaired by the manipulation of the N-terminus. This could be improved by applying another acyltransferase, such as isoprenyltransferases. The technological advances in cell-free systems described here should facilitate production of proteins that require post-translational modifications and production of functional liposomes via lipidation of soluble proteins.

## Materials and Methods

### Materials and Chemicals

Histidine tagged-TEV protease (glycerol-free) and nanodisc (MSP1E3D1-His/DMPG and MSP1E3D1-His/DMPC) were purchased from Fujifilm and Cube Biotech, respectively. Myristoyl-CoA and palmitoyl-CoA were purchased from SIGMA-Aldrich. 1-Palmitoyl-2-oleoyl-*sn*-glycero-3-phosphocholine (POPC) and 1-palmitoyl-2-oleoylphosphatidylglycerol (POPG) were purchased from Avanti Polar. Rhodamine-DHPE, OptiPrep, and 1,2-distearoyl-*sn*-glycero-3-phosphoethanolamine-*N*-[amino­(polyethylene glycol)-2000] (DSPE-PEG2000) were purchased from Nakarai tesque. Ficoll PM70 was purchased from Cytiva. Anti 6 × Histidine, monoclonal antibody­(9C11), peroxidase conjugated (Cat. No. 010-23181) was purchased from Fujifilm.

### Preparation of Template DNA for Cell-Free Protein Synthesis

DNA sequences for cell-free protein synthesis were designed using CodHonEditor^31^ based on codon usage and synthesized by Eurofins genomics and GenScript. All template DNAs were prepared by PCR using synthetic DNA (Table S1) and primers described in Table S2. The amplified DNAs, which were identified by agarose gel electrophoresis, were purified with a PCR purification kit, and their concentrations were determined by measurement of absorbance at 260 nm. A hNMT lacking 1–80 amino acids at the N-terminus was used as NMT, as described in a previous report.^22^

### Cell-Free Reactions

The substrate proteins were synthesized using PURE*frex*2.1 (GeneFrontier, Japan) according to the manufacturer’s instructions, supplemented with 1 ng/μL (final concentration, f.c.) template DNA and 50 ng/μL (f.c.) TEV protease. A 20 μL reaction mixtures was incubated at 37 °C for 4 h. The resulting mixture was centrifuged at 20,000*g*, 30 min at 4 °C, and the supernatants were collected that will be used as substrate proteins. For the NMT synthesis, a DnaK chaperone mix, containing DnaK, DnaJ, and GrpE (GeneFrontier, Japan), was also supplemented. A 20 μL reaction mixture was incubated at 30 °C for 24 h, and then the supernatant was collected after the centrifugation. For the lipidation reaction, in general, 5–7 μM substrate protein, 0.5 μM NMT, and 100 μM myristoyl-CoA or palmitoyl-CoA were mixed and filled up 20 μL with pure water. The resulting mixtures were incubated at 30 °C for the indicated hours.

### Protein Lipidation in GUVs

Protein lipidation inside GUVs was performed as described previously.^22^ Briefly, the lipidation reaction mixture containing 200 mM sucrose and 12% (w/v) Ficoll PM70 was encapsulated inside GUVs by the method of Shimane et al.^32^ The lipid composition of POPC 80 mol % and POPG 20 mol % was employed for GUV formation. The internal lipidation reaction was performed up to 20 h.

### Nanodisc Localization Assay

Cell-free synthesized HisTEV-Goα­(S)-sfGFP was purified using a Ni-Sepharose 6 Fast Flow (Cytiva). Purified substrate protein was digested using TEV protease during dialysis and purified again using Ni-Sepharose 6 Fast Flow to remove the N-terminal fragment of the synthesized proteins and TEV protease. A 7 μM purified-and-digested substrate protein was mixed with 0.5 μM NMT, 100 μM myristoyl-CoA or palmitoyl-CoA, and 10 μM nanodisc (MSP1E3D1-His/DMPG or MSP1E3D1-His/DMPC), followed by incubation at 30 °C for 24 h. His-tagged nanodisc was isolated using Ni-Sepharose 6 Fast Flow, and the fluorescence in the fraction of the isolated nanodisc was measured using a Varioskan LUX multimode microplate reader (Thermo).

### Liposome Localization Assay

Liposomes were prepared as described previously^33^ with POPC/POPG/DSPE-PEG2000/rhodamine-DHPE (68:30:2:0.5 mol %). Briefly, a mixture of phospholipids dissolved in chloroform was evaporated in a flask and hydrated with 50 mM Hepes-KOH (pH 7.6) to obtain a 40 mg/mL liposome solution. The liposome size was homogenized by using a microextruder equipped with a 100 nm pore-size polycarbonate membrane. For the liposome localization assay, 2 mg/mL (f.c.) liposome was supplied into 40 μL of the lipidation reaction. After incubation at 37 °C for 3 h, the resulting mixture was analyzed by the flotation assay.

### Flotation Assay

A 40 μL cell-free reaction mixture-containing liposomes was mixed with 80 μL of 60% (w/v) OptiPrep solution and transferred into centrifuge tubes (7 × 20 mm, Beckman Coulter REF: 343775). An 80 μL portion of 25% (w/v) OptiPrep solution, which was prepared by diluting the 60% solution with 100 mM Hepes-KOH (pH 7.5), was overlaid, and then 60 μL of 0% solution (i.e., 100 mM Hepes-KOH (pH 7.5)) was followed. The resulting OptiPrep gradient solutions were ultracentrifuged at 197,000*g* for 2 h at 4 °C using a TLA-100 fixed angle rotor (Beckman Coulter). After the ultracentrifugation, 40 μL of the OptiPrep solutions were collected from the top four times, and 100 μL of the bottom solution was collected. The remaining precipitates were dissolved in 40 μL of 100 mM Hepes-KOH (pH 7.5) buffer. 10 μL of the solutions from each fraction was transferred into a 96-well black plate, and the fluorescent signals of rhodamine-DHPE were measured. Additionally, 8 μL of each fraction was analyzed by SDS-PAGE after mixing with loading buffer and boiling at 97 °C for 5 min, followed by Western blotting using anti-6 × histidine monoclonal antibody conjugating peroxidase.

To estimate the number of VHH protein on a liposome, protein concentration was measured by Western blotting with defined standards, and the number of liposome particles per μL was measured by dynamic light scattering (Malvern Panalytical), and then protein concentration was divided by the liposome concentration to estimate the number of VHH proteins per liposome.

### Microscopic Observation

Giant vesicles were observed by using Nikon A1R confocal microscopy system equipped with a 488 nm laser. In all cases, the images were captured as a set with DIC microscopy. The membrane localization percentage of the lapidated substrate GFPs was estimated by the plot profile measurement of fluorescent vesicles along the equator using ImageJ software.

### Cell Assay

Cell-free lipidation reaction was first performed in a 40 μL mixture, which consists of 17 μL of PURE reaction solution in which NMT synthesized, 3 μL of PURE reaction solution in which Goα­(S)-VHH synthesized, 1 μL of 2 mM palmitoyl-CoA (f.c. 50 μM), 2 μL of 40 mg/mL liposome (POPC 68%, POPG 30%, DSPE-PEG2000 2% (mol.), and rhodamine B-DHPE), and 17 μL of 10 mM Hepes-KOH (pH 7.5). Reaction was carried out at 37 °C for 3 h. The cells expressing HER2 (BT474 cell line) and control cells lacking HER2 (T47D cell line) were used for the assay. The cultivated cells were washed with 100 μL D-PBS (+) three times, then reacted with 100 μL VHH-displaying liposomes, of which 5 μL cell-free reaction mixture was diluted with PBS. After 30 min incubation within CO_2_ incubator at 37 °C, the cells were washed again as same as above. The resulting cells were observed within 100 μL of D-PBS (+) by Nikon A1R confocal microscopy system equipped with a 561 nm laser.

### Detection of the N-Terminal Peptides by LC–MS/MS

The detection of N-terminal peptides by LC–MS/MS from in-gel digested samples was performed according to the method reported previously.^22^ Briefly, the excised gel bands after SDS-PAGE were destained, dehydrated, and evaporated. After that, the dried gels were soaked with 50 mM ammonium bicarbonate solution containing 12.5 μg/mL Trypsin Gold (Promega, Cat. No. V5280) and incubated at 37 °C overnight for digestion. After incubation, the digested peptides were extracted with a 50% acetonitrile solution. The solution was dried and desalted using a GL-Tip SDB instrument (GL Sciences, Japan).

The in-solution digestion was performed using the following procedure. The samples after the lipidation reaction were mixed with 8 M urea and 50 mM ammonium bicarbonate solution in a 1:3 ratio. The samples were reduced by adding dithiothreitol at a final concentration of 10 mM and incubated at room temperature for 30 min. Then, the samples were alkylated by adding iodoacetamide at a final concentration of 50 mM and incubated at room temperature for 30 min in the dark. After reduction and alkylation, the samples were diluted 1.5-fold with 50 mM ammonium bicarbonate and digested by adding Lys-C protease (Fujifilm Wako, Cat. No. 125-05061) and incubated for >2 h at room temperature. After 3-fold dilution with 50 mM ammonium bicarbonate, the samples were further digested by adding Trypsin Gold (Promega, Cat. No. V5280) and incubated at 37 °C overnight. The solution was dried and desalted using a GL-Tip SDB (GL Sciences, Japan).

The LC–MS/MS measurements were conducted with a Q-Exactive tandem mass spectrometer and an Easy-nLC 1000 nano HPLC system (Thermo Fisher Scientific). MS/MS data were acquired in data-dependent acquisition (DDA) mode controlled by Xcalibur 4.0 software (Thermo Fisher Scientific). The detailed settings of nanoLC and DDA measurement were the same as those reported previously.^22^ For the in-solution digested samples, the gradient condition was changed to a 5–55% linear acetonitrile gradient over 50 min in the presence of 0.1% formic acid.

The MS/MS data were analyzed with Proteome Discoverer (version 2.4 or 3.1, Thermo Fisher Scientific) bundled with the Sequest HT search engine for peptide annotation. For the detection of post-translational modifications, myristoylation (+210.1984 Da, C_14_H_26_O) and palmitoylation (+238.2297 Da, C_16_H_30_O) were defined as dynamic modifications on Gly residue at N-terminus. Generation of the extracted ion chromatogram of the corresponding *m*/*z* and quantification were performed using Skyline software (version 24.1).^34^

## Supplementary Material



## Notes

Microscopy images of giant vesicles performing lipidation of protein inside and those membrane localization efficiency, nonlocalization of myristoylated protein onto liposomes, LC–MS/MS analysis for the lipidated proteins, localization of palmitoylated proteins containing polyarginine (R3) onto liposomes, schematics of the DNA constructs coding VHH antibody used for cell-free lipidation, gel image of flotation assay, microscopy image of breast cancer cells expressing HER2 and treated with liposomes carrying lipidated VHH antibodies, and DNA sequences of all genes and primers (PDF)
