# Supplementary material for: Lipid Modification and Membrane Localization of Proteins in Cell-Free System
Source: ACS Synth Biol. 2025 Jun 19;14(7):2729–38. doi: 10.1021/acssynbio.5c00155 (PMC12281620; doi:10.1021/acssynbio.5c00155)
Supplement: Supplementary file 1 [file sb5c00155_si_001.pdf]

Supporting Information

Lipid modification and membrane localization of proteins in cell-free system

Rena Matsumoto[1], Tatsuya Niwa[2], Kaori Kuno[3], Yasuhiro Shimane[3], Yutetsu Kuruma[3]*, Takashi Kanamori[1]*

[1] GeneFrontier Corporation, 273-1 Kashiwa, Kashiwa, Chiba 277-0005, Japan

[2] Cell Biology Center, Institute of Integrated Research, Institute of Science Tokyo, Yokohama, Kanagawa 226-8501, Japan

[3] Institute for Extra-cutting-edge Science and Technology Avant-garde Research (X-star), Japan Agency for Marine-Earth Science and Technology (JAMSTEC), 2-15 Natsushima-cho, Yokosuka, Kanagawa 237-0061, Japan

*To whom correspondence should be addressed. E-mail: T.K., kanamori@genefrontier.com, or Y.K., ykuruma@jamstec.go.jp

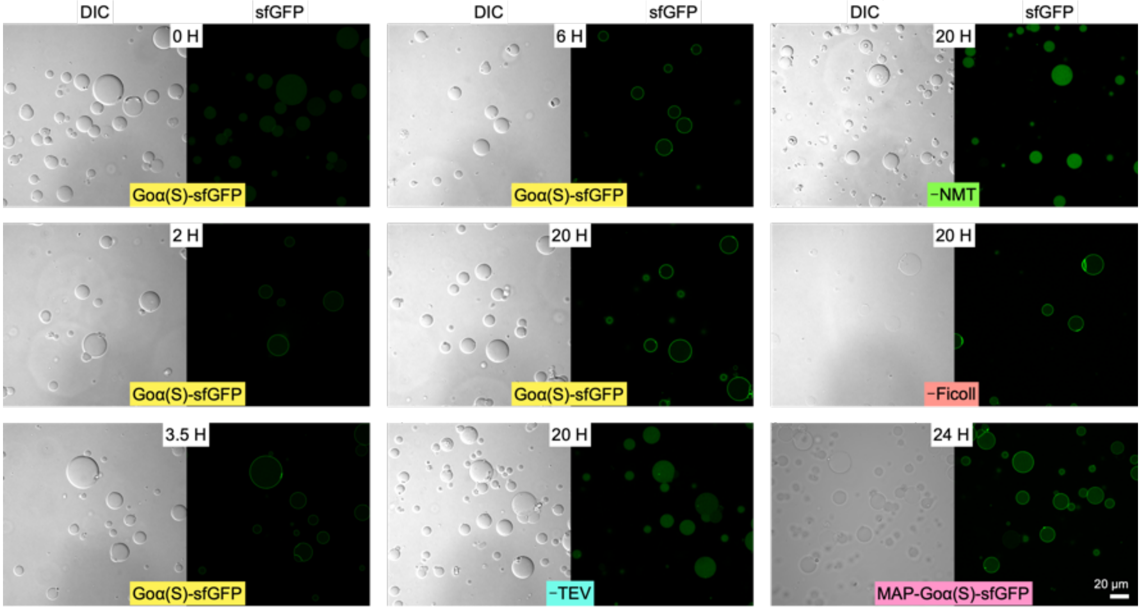

Figure S1. Lipid modification of protein inside giant vesicles. Goα(S)-sfGFP were myristoylated inside giant vesicles with the controls of without TEV protease (−TEV), N-myristoyltransferase (−NMT), and Ficoll PM70 (−Ficoll). Additionally, the construct using methionine aminopeptidase (MAP) instead of TEV was also tested for comparison (MAP-Goα(S)-sfGFP). Vesicles were observed by confocal microscopy at the indicated time with the set of differential interference construct (DIC) and 488 nm laser. Bar: 20 µm.

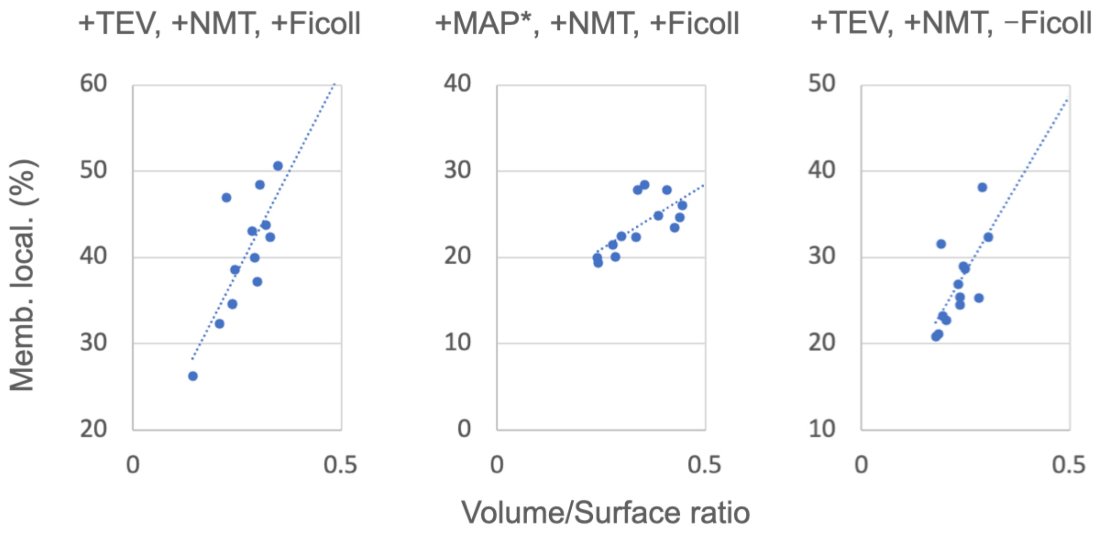

Figure S2. Correlation between membrane localization efficiency and the ratio of volume per surface. Membrane localization efficiency of myristoylated Goα(S)-sfGFP and the diameter of vesicles were measured by ImageJ. *MAP (methionine aminopeptidase) was used to trim the precursor of Goα(S)-sfGFP instead of TEV. More than 10 vesicles were analyzed for each sample.

Reaction mixture

	+NMT (μL)	−NMT (μL)
Goα(S)-sfGFP	3	3
NMT	20	0
Myr-CoA	1	1
Liposome*	2	2
Buf†	7	17
H_2O	7	17
Total	40	40

↓ 37 ˚C, 2 h

↓ Flotation assay, 197,000 x G, 2 h, 4 ˚C

↓ Fractionation

↓ SDS-PAGE

*POPC 85%, POPG 8%, Chol 5%, PEG2K-PE 2%, Rhodamin-PE

†PURE system buffer

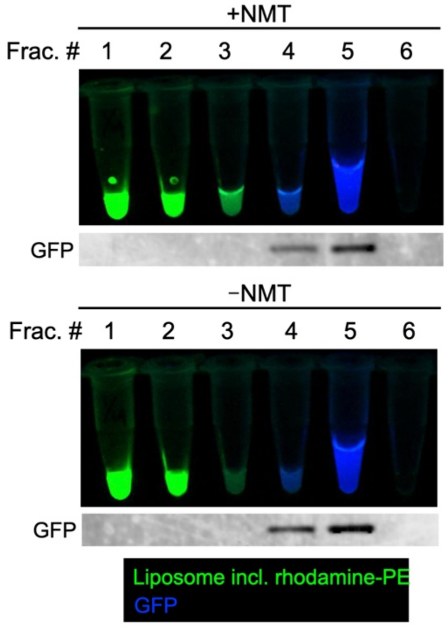

Figure S3. Localization of myristoylated protein onto liposomes. Myristoylation of Goα(S)-sfGFP were performed in the presence of liposomes in the set of NMT + and − for 2 hours at 37°C. resulting liposomes were fractionated by flotation assay and the localization of Goα(S)-sfGFP were evaluated by SDS-PAGE. Fraction (Frac.) 1 and 6 indicate the top and bottom of OptiPrep density gradient (see Fig. 4A and Materials and Methods).

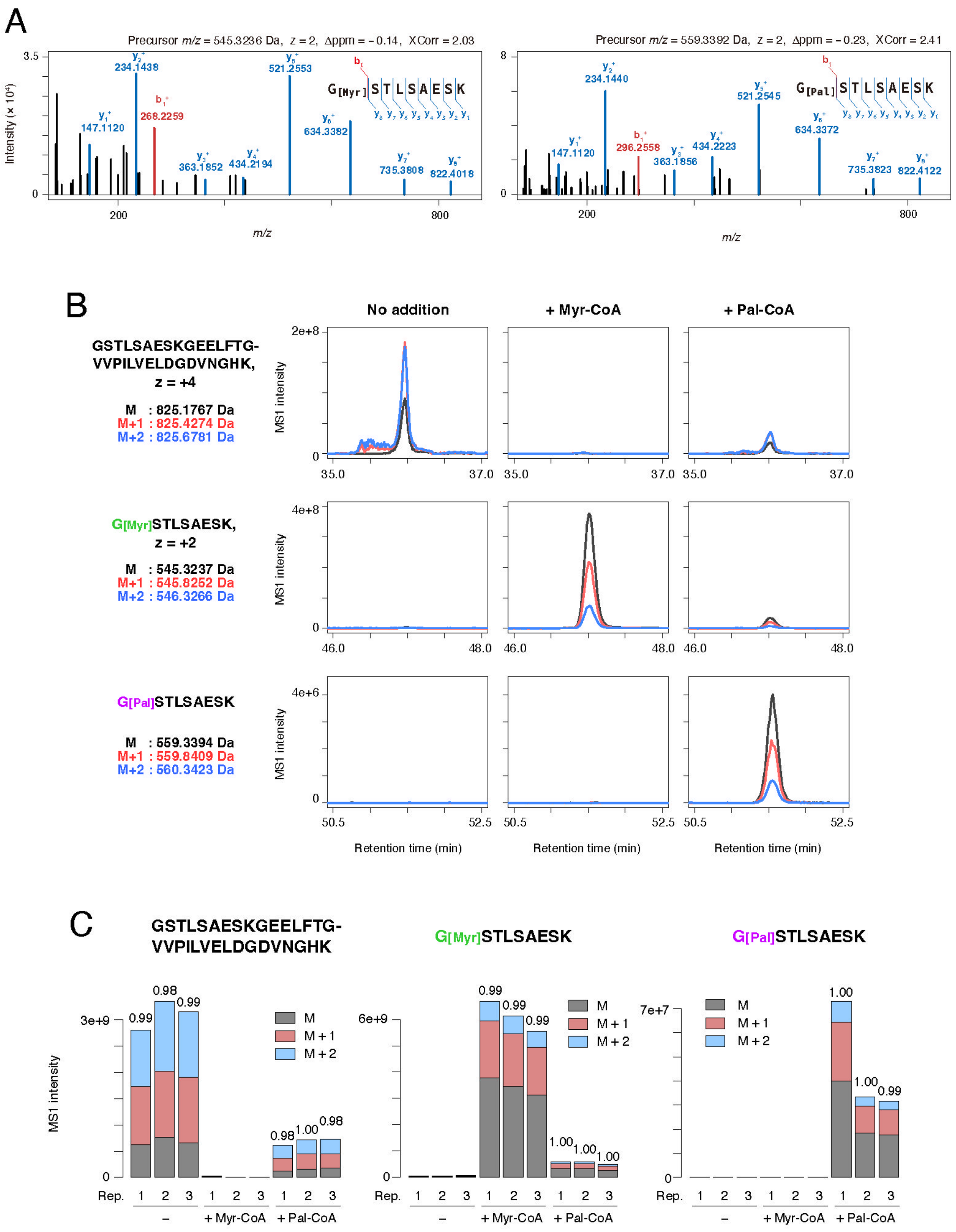

Figure S4. Confirmation of myristoylation and palmitoylation by LC-MS/MS analysis. (A) MS/MS spectra of the myristoylated and palmitoylated peptides detected by LC-MS/MS analysis. The peaks

of the b- and y-fragment ions were shown in red and blue, respectively. The annotation of the fragment ions and the peptide annotation parameters were obtained from the analysis by Proteome Discoverer (ver. 3.1). (B) Extracted ion chromatograms of the detected peptides. For simplicity, only the signals from one technical replicate were shown. Gray, red, and blue lines indicate the chromatogram of M, M+1, and M+2 signals, respectively. (C) MS1 signal intensities of the detected peptides. Replicates 1, 2, and 3 indicate the technical replicates on LC-MS/MS measurement. The numbers above the bar indicated an isotope dot product (idotp) score calculated by Skyline software. We confirmed that a very similar trend was observed in another replicate set.

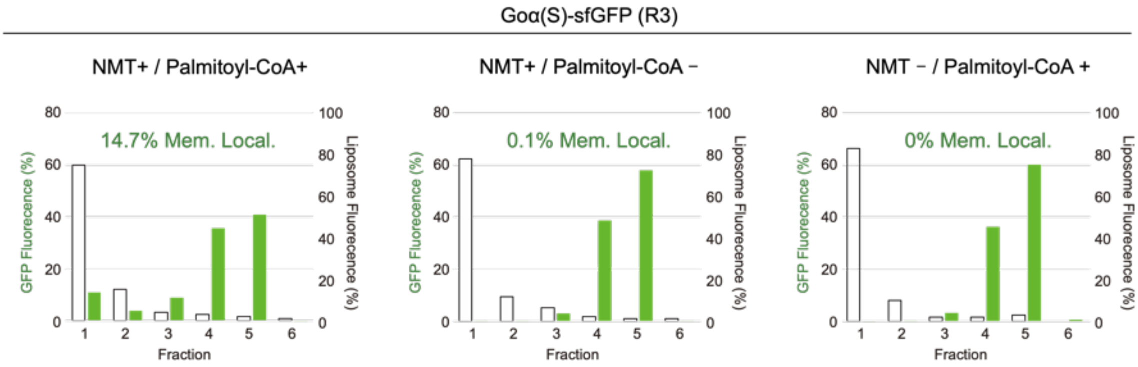

Figure S5. Localization of Goα(S)-sfGFP containing polyarginine (R3) onto liposomes. Palmitoylation of Goα(S)-sfGFP(R3) (green color bar) were performed in the presence of liposomes (white color bar) with the controls of Palmitoyl-CoA– and NMT–. Membrane localization efficiency combing fraction 1 and 2 were indicated within the graphs.

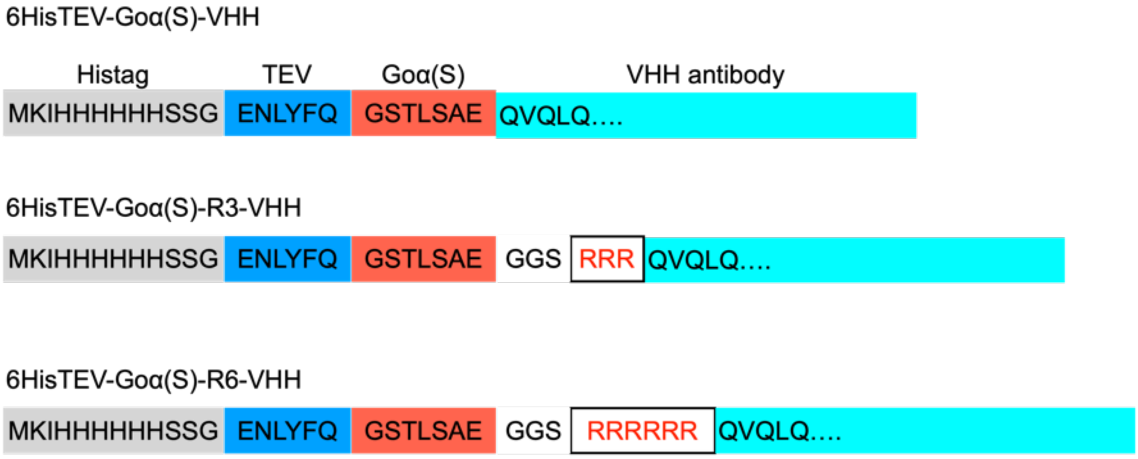

Figure S6. Constructs of polyarginine-containing VHH antibody. As same as Goα(S)-sfGFP constructs, a VHH antibody gene was located to the downstream of Histag, TEV, and Goα(S). A GGS linker and polyarginine RRR or RRRRRR were inserted between Goα(S) and VHH for 6HisTEV-Goα(S)-R3-VHH or 6HisTEV-Goα(S)-R6-VHH constructs, respectively.

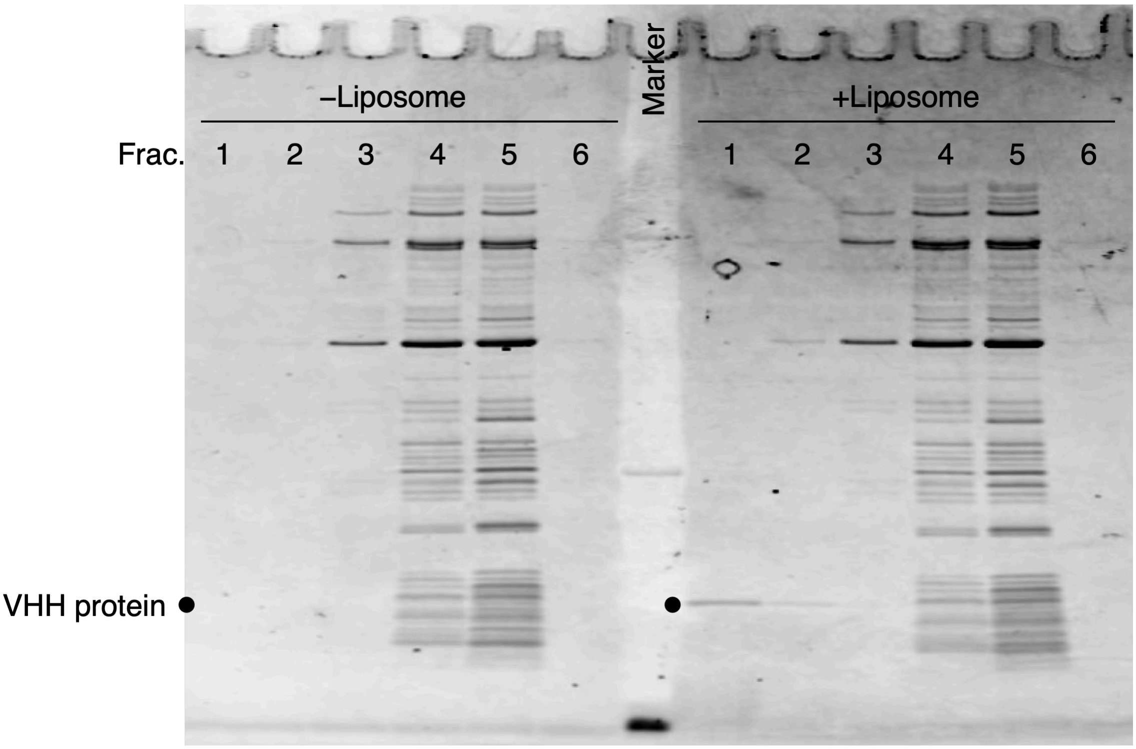

Figure S7. Flotation assay of the palmitoylated-VHH antibody (2Rs15d) containing six polyarginines. The fractionated samples were analyzed by SDS-PAGE and stained by Oriole. The position of VHH protein is indicated with filled circle.

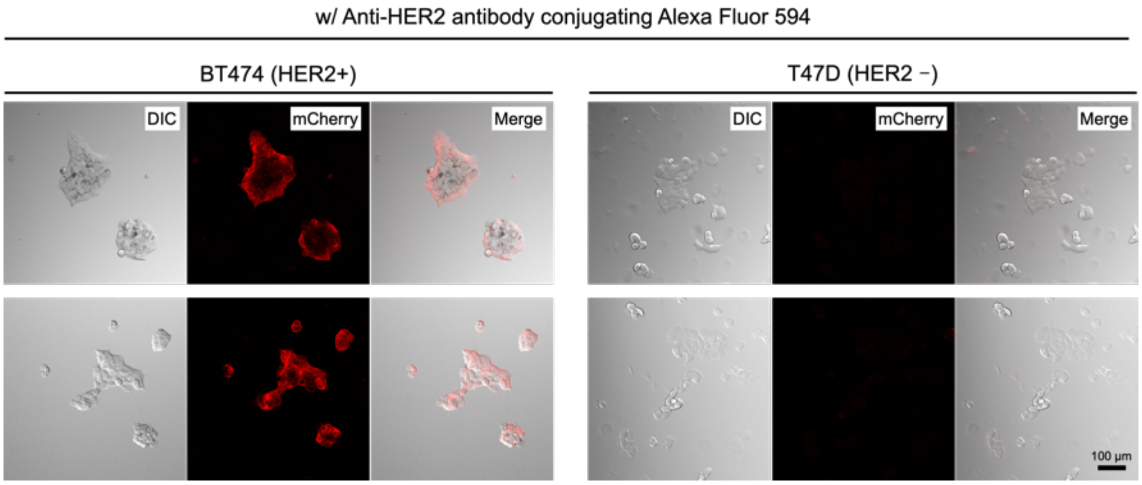

Figure S8. Verification of HER2 expression in BT474 cell line. The presence of HER2 was detected by using of anti-HER2 antibody-conjugating Alexa Fluor 594. The control cells which do not express HER2 (T47D) were also tested. Cells were observed by Nikon A1R confocal microscopy with the set of differential interference construct (DIC) and 561 nm laser. Bar: 100 μm.

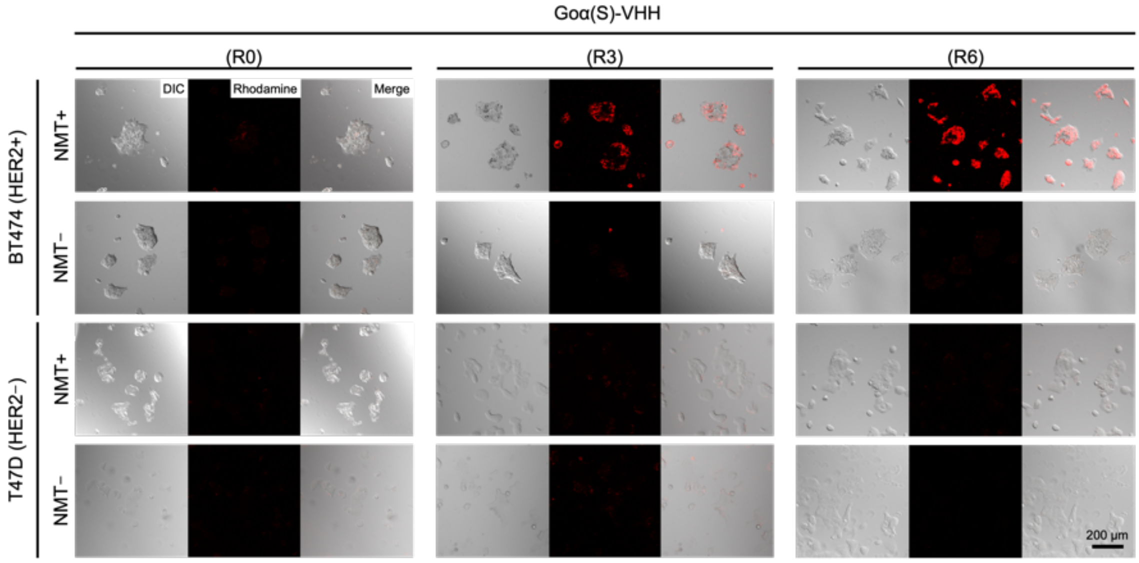

Figure S9. Specific binding of VHH-presenting liposomes to the target cells. Goα(S)-VHH(R0, R3, or R6) were palmitoylated and localized onto liposomes containing rhodamine-DHPE. The resulting liposomes were mixed with the cells of HER2 positive (HER2+) or negative (HER2−). For each case, negative control lacking NMT− were also analyzed. Cells were observed by Nikon A1R confocal microscopy with the set of differential interference construct (DIC) and 561 nm laser. Bar: 200 µm.

Table S1. DNA sequences of genes used in this study.

6HisTEV-Goα-sfGFP
GAAATTAATACGACTCACTATAGGGAGACCACAACGGTTTCCCTCTAGAAATAATTTTG<br>TTTAACTTTAAG**AAGGAG**ATATACCAATGAAAATTCATCATCATCATCATCATTCTTCTG<br>GTGAAAACTTATACTTTCAGGGAAGTACATTAAGCGCAGAATCTAAAGGTGAAGAATT<br>ATTTACTGGTGTTGTGCCGATCCTGGTCGAACTGGACGGTGATGTGAATGGGCATAA<br>ATTCTCGGTTCGGGGCGAAGGAGAGGGTGACGCAACTAACGGCAAACTTACCCTCA<br>AGTTTATTTGTACCACAGGTAAACTGCCAGTCCCGTGGCCCACGCTGGTGACCACTT<br>TGACCTACGGCGTACAGTGCTTCAGCCGCTATCCGGATCACATGAAACGTCATGATT<br>TCTTCAAATCAGCGATGCCTGAAGGGTATGTTCAGGAACGCACGATTAGCTTTAAAG<br>ACGATGGCACCTACAAGACACGTGCCGAGGTGAAATTTGAAGGTGATACGTTAGTCA<br>ATCGCATCGAACTGAAAGGCATTGACTTCAAAGAGGATGGAAACATCCTGGGTCATA<br>AGCTGGAATATAACTTTAATTCTCACAACGTGTACATTACCGCTGACAAACAAAAGAAT<br>GGCATCAAAGCGAACTTCAAGATTCGTCATAATGTTGAAGATGGGTCCGTACAGCTT<br>GCAGATCACTATCAGCAAAACACTCCGATCGGTGACGGCCCAGTGCTCCTGCCGGA<br>TAATCATTACTTGAGTACCCAGTCGGTCTTAAGCAAAGATCCGAACGAGAAACGCGA<br>CCACATGGTTCTGCTGGAATTTGTGACGGCCGCGGGTATTACCCATGGCATGGATGA<br>ACTGTATAAAGGATCTGACTATAAAGATGACGATGACAAATAATGAATAACTAATCC
6HisTEV -Goα-3R-sfGFP
GAAATTAATACGACTCACTATAGGGAGACCACAACGGTTTCCCTCTAGAAATAATTTTG<br>TTTAACTTTAAG**AAGGAG**ATATACCAATGAAAATTCATCATCATCATCATCATTCTTCTG<br>GTGAAAACTTATACTTTCAGGGAAGTACATTAAGCGCAGAAGGTGGCAGTCGTCGCC<br>GTTCTAAAGGTGAAGAATTATTTACTGGTGTTGTGCCGATCCTGGTCGAACTGGACG<br>GTGATGTGAATGGGCATAAATTCTCGGTTCGGGGCGAAGGAGAGGGTGACGCAACT<br>AACGGCAAACTTACCCTCAAGTTTATTTGTACCACAGGTAAACTGCCAGTCCCGTGG<br>CCCACGCTGGTGACCACTTTGACCTACGGCGTACAGTGCTTCAGCCGCTATCCGGA<br>TCACATGAAACGTCATGATTTCTTCAAATCAGCGATGCCTGAAGGGTATGTTCAGGAA<br>CGCACGATTAGCTTTAAAGACGATGGCACCTACAAGACACGTGCCGAGGTGAAATTT<br>GAAGGTGATACGTTAGTCAATCGCATCGAACTGAAAGGCATTGACTTCAAAGAGGAT<br>GGAAACATCCTGGGTCATAAGCTGGAATATAACTTTAATTCTCACAACGTGTACATTAC<br>CGCTGACAAACAAAAGAATGGCATCAAAGCGAACTTCAAGATTCGTCATAATGTTGAA<br>GATGGGTCCGTACAGCTTGCAGATCACTATCAGCAAAACACTCCGATCGGTGACGG<br>CCCAGTGCTCCTGCCGGATAATCATTACTTGAGTACCCAGTCGGTCTTAAGCAAAGA<br>TCCGAACGAGAAACGCGACCACATGGTTCTGCTGGAATTTGTGACGGCCGCGGGTA<br>TTACCCATGGCATGGATGAACTGTATAAAGGATCTGACTATAAAGATGACGATGACAA<br>ATAATGAATAACTAATCC

6HisTEV -Goα-6R-sfGFP
GAAATTAATACGACTCACTATAGGGAGACCACAACGGTTTCCCTCTAGAAATAATTTTG
TTTAACTTTAAG**AAGGAG**ATATACCAATG*AAAATTCATCATCATCATCATCATTCTTCTG*
GTGAAAACTTATACTTTCAGGGAAGTACATTAAGCGCAGAAGGTGGCAGTCGTCGCC
GTCGTCGCCGCTCTAAAGGTGAAGAATTATTTACTGGTGTTGTGCCGATCCTGGTCG
AACTGGACGGTGATGTGAATGGGCATAAATTCTCGGTTCGGGGCGAAGGAGAGGGT
GACGCAACTAACGGCAAACTTACCCTCAAGTTTATTTGTACCACAGGTAAACTGCCA
GTCCCGTGGCCCACGCTGGTGACCACTTTGACCTACGGCGTACAGTGCTTCAGCCG
CTATCCGGATCACATGAAACGTCATGATTTCTTCAAATCAGCGATGCCTGAAGGGTAT
GTTCAGGAACGCACGATTAGCTTTAAAGACGATGGCACCTACAAGACACGTGCCGA
GGTGAAATTTGAAGGTGATACGTTAGTCAATCGCATCGAACTGAAAGGCATTGACTT
CAAAGAGGATGGAAACATCCTGGGTCATAAGCTGGAATATAACTTTAATTCTCACAAC
GTGTACATTACCGCTGACAAACAAAAGAATGGCATCAAAGCGAACTTCAAGATTCGT
CATAATGTTGAAGATGGGTCCGTACAGCTTGCAGATCACTATCAGCAAAACACTCCG
ATCGGTGACGGCCCAGTGCTCCTGCCGGATAATCATTACTTGAGTACCCAGTCGGTC
TTAAGCAAAGATCCGAACGAGAAACGCGACCACATGGTTCTGCTGGAATTTGTGACG
GCCGCGGGTATTACCCATGGCATGGATGAACTGTATAAAGGATCTGACTATAAAGATG
*ACGATGACAAATAA*TGAATAACTAATCC

NMT
GAAATTAATACGACTCACTATAGGGAGACCACAACGGTTTCCCTCTAGAAATAATTTTG
TTTAACTTTAAG**AAGGAG**ATATACCAATG*aattcattacctgcaGAGCGCATCCAAGAAATCC*
AGAAAGCCATTGAACTGTTCTCTGTAGGTCAGGGCCCGGCTAAAACCATGGAAGAG
GCGTCCAAGCGTAGTTACCAGTTTTGGGATACACAACCGGTTCCGAAATTAGGTGAA
GTGGTAAACACTCACGGCCCAGTTGAACCGGACAAAGACAACATCCGCCAGGAGCC
TTATACCCTTCCGCAAGGATTCACTTGGGATGCTCTGGACCTGGGGGATAGGGGCG
TCCTGAAAGAATTGTACACGCTCCTGAACGAAAATTACGTTGAAGACGATGACAACAT
GTTCCGATTTGATTATAGCCCGGAGTTCCTGCTGTGGGCACTTCGTCCCCCAGGTTG
GCTGCCGCAATGGCATTGTGGCGTACGTGTGGTTTCTTCCCGCAAGTTAGTAGGTTT
CATCTCAGCTATTCCTGCCAACATCCACATTTACGACACCGAAAAGAAAATGGTTGAA
ATCAACTTTCTGTGCGTGCATAAGAAACTGCGTTCTAAACGCGTCGCGCCGGTTCTG
ATCCGTGAGATTACTCGGAGAGTACACCTCGAAGGAATCTTCCAGGCAGTGTATACC
GCTGGTGTTGTACTGCCGAAACCGGTTGGCACTTGTCGTTACTGGCACCGCAGCTT
GAATCCACGTAAGCTGATCGAAGTCAAATTTTCGCATCTTTCTCGCAACATGACGATG
CAGCGTACCATGAAACTGTACCGTCTGCCTGAAACTCCGAAAACCGCCGGATTACG
CCCCATGGAGACAAAGGATATTCCGGTGGTTCACCAGCTGCTGACTCGTTATCTCAA
ACAATTCCATCTGACGCCGGTAATGTCCCAGGAAGAAGTTGAGCACTGGTTCTACCC

ACAGGAAAACATCATCGACACCTTTGTGGTAGAAAACGCTAATGGGGAAGTTACTGA<br>*TTTCCTTAGTTTCTACACCCTGCCTAGCACTATTATGAACCACCCGACGCATAAATCTC*<br>*TGAAAGCAGCCTATTCCTTTTACAACGTCCACACCCAAACTCCGCTGTTGGACCTGA*<br>*TGAGCGATGCTTTAGTTCTGGCGAAGATGAAAGGCTTCGACGTGTTCAACGCACTC*<br>*GATCTGATGGAGAATAAAACCTTTCTTGAAAAACTGAAGTTCGGTATCGGCGACGGT*<br>*AACCTGCAGTACTATCTGTACAACTGGAAATGCCCGTCTATGGGCGCTGAAAAAGTA*<br>*GGTCTGGTTCTGCAG*TAATGAATAACTAATCC
6HisTEV-Goa-VHH
GAAATTAATACGACTCACTATAGGGAGACCACAACGGTTTCCCTCTAGAAATAATTTTG<br>TTTAACTTTAAG**AAGGAG**ATATACCAATG*AAAATTCATCATCATCATCATCATTCTTCTG*<br>*GTGAAAACTTATACTTTCAGGGAagtACATTAAGCGCAGAACAGGTTCAGCTCCAGGA*<br>*ATCCGGTGGCGGTTCAGTGCAAGCCGGAGGTTCTCTGAAACTTACCTGTGCTGCGA*<br>*GCGGCTACATCTTCAACTCGTGCGGTATGGGGTGGTATCGCCAGTCTCCGGGCCGT*<br>*GAACGTGAACTGGTATCCCGCATTAGTGGTGATGGCGACACTTGGCACAAAGAGAG*<br>*CGTTAAAGGTCGTTTTACCATCTCTCAGGATAATGTCAAGAAAACTCTGTACCTGCAA*<br>*ATGAACTCCCTGAAACCGGAAGACACGGCAGTTTACTTCTGTGCTGTGTGCTATAAC*<br>*CTGGAAACCTACTGGGGCCAGGGTACTCAGGTAACCGTTAGCTCT*TAATGAATAACT<br>AATCC
6HisTEV-Goa-R3-VHH
GAAATTAATACGACTCACTATAGGGAGACCACAACGGTTTCCCTCTAGAAATAATTTTG<br>TTTAACTTTAAG**AAGGAG**ATATACCAATG*AAAATTCATCATCATCATCATCATTCTTCTG*<br>*GTGAAAACTTATACTTTCAGGGAAGTACATTAAGCGCAGAAGGTGGCAGTCGTCGCC*<br>*GTCAGGTTCAGCTCCAGGAATCCGGTGGCGGTTCAGTGCAAGCCGGAGGTTCTCT*<br>*GAAACTTACCTGTGCTGCGAGCGGCTACATCTTCAACTCGTGCGGTATGGGGTGGT*<br>*ATCGCCAGTCTCCGGGCCGTGAACGTGAACTGGTATCCCGCATTAGTGGTGATGGC*<br>*GACACTTGGCACAAAGAGAGCGTTAAAGGTCGTTTTACCATCTCTCAGGATAATGTC*<br>*AAGAAAACTCTGTACCTGCAAATGAACTCCCTGAAACCGGAAGACACGGCAGTTTAC*<br>*TTCTGTGCTGTGTGCTATAACCTGGAAACCTACTGGGGCCAGGGTACTCAGGTAACC*<br>*GTTAGCTCT*TAATGAATAACTAATCC
6HisTEV-Goa-R6-VHH
GAAATTAATACGACTCACTATAGGGAGACCACAACGGTTTCCCTCTAGAAATAATTTTG<br>TTTAACTTTAAG**AAGGAG**ATATACCAATG*AAAATTCATCATCATCATCATCATTCTTCTG*<br>*GTGAAAACTTATACTTTCAGGGAAGTACATTAAGCGCAGAAGGTGGCAGTCGTCGCC*<br>*GTCGTCGCCGCCAGGTTCAGCTCCAGGAATCCGGTGGCGGTTCAGTGCAAGCCGG*<br>*AGGTTCTCTGAAACTTACCTGTGCTGCGAGCGGCTACATCTTCAACTCGTGCGGTAT*<br>*GGGGTGGTATCGCCAGTCTCCGGGCCGTGAACGTGAACTGGTATCCCGCATTAGTG*

*GTGATGGCGACACTTGGCACAAAGAGAGCGTTAAAGGTCGTTTTACCATCTCTCAGG ATAATGTCAAGAAAACTCTGTACCTGCAAATGAACTCCCTGAAACCGGAAGACACGG CAGTTTACTTCTGTGCTGTGTGCTATAACCTGGAAACCTACTGGGGCCAGGGTACTC AGGTAACCGTTAGCTCT*TAATGAATAACTAATCC
T7 promoter, **Ribosome binding domain**, Initial codon, Stop codon, *Open reading frame*

Table S2. DNA sequences of primers used in this study.

Primer name	Sequence	Length	Remarks
T7Pro_F	GAAATTAATACGACTCACTATAGGGAGACCACAACGG	37 nt	Forward primer for all
FLAG_R	GGATTAGTTATTCATTATTTGTCATCGTCATCTTTATAGTCAGATCC	47 nt	Reverse primer for substrate proteins
NMT_R	GGATTAGTTATTCATTACTGCAGAACCAGACCTACTTTTTC	41 nt	Reverse primer for NMT
